# Metabolite‐based resistance in wheat varieties to aphid virus vectors: progress and future opportunities

**DOI:** 10.1002/ps.8780

**Published:** 2025-03-21

**Authors:** Alexander N Borg, József Vuts, John C Caulfield, Michael A Birkett

**Affiliations:** ^1^ Protecting Crops and the Environment Rothamsted Research Harpenden UK; ^2^ Division of Plant and Crop Sciences The University of Nottingham, Sutton Bonington Campus Loughborough UK

**Keywords:** aphid resistance, natural products, primary metabolites, *Rhopalosiphum padi*, *Sitobion avenae*, wheat, Triticum

## Abstract

Cereal aphids, *Sitobion avenae* and *Rhopalosiphum padi*, cause severe yield loss in wheat crops as a consequence of direct feeding damage and acting as vectors for Barley Yellow Dwarf Virus (BYDV). Insecticides have commonly been used to control these pests, but the advent of insecticide resistance spreading across aphid populations and the push to reduce insecticide use means that new approaches to control aphid populations are required. Wheat varieties with metabolite‐based aphid resistance have been identified, suggesting that they could be developed as an alternative to insecticides. Resistance induced by natural products (metabolites) include volatile organic compound‐mediated (antixenotic) and development‐modifying (antibiotic) processes. Full characterisation of these resistance mechanisms is still required, and associated challenges, such as the influence of biotic and abiotic interactions, need to be addressed prior to their implementation into integrated pest management (IPM) or engineered into modern elite wheats. In this review, current literature on metabolite‐based *S. avenae* and *R. padi* resistance in wheat is discussed, outlining current knowledge gaps and challenges, and highlighting the future work required. © 2025 The Author(s). *Pest Management Science* published by John Wiley & Sons Ltd on behalf of Society of Chemical Industry.

## Graphical abstract

*Sitobion avenae* and *Rhopalosiphum padi* are major pests on wheat. Natural product‐based aphid resistance holds promise for the sustainable control of these pests. The current literature is discussed, highlighting knowledge gaps and challenges that need addressing.

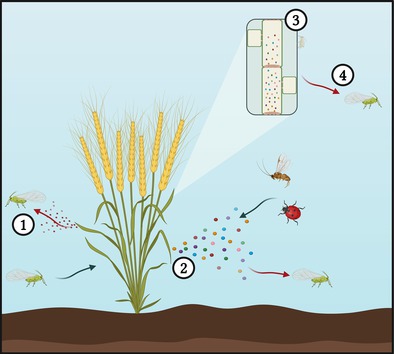

## 1 INTRODUCTION

Wheat, *Triticum aestivum* L. (Poaceae), is a globally important staple food crop (FAO; https://www.fao.org/faostat). Aphids (Hemiptera: Aphididae) are the most economically important pest insects on wheat,^1^ causing damage either by phloem‐feeding or virus transmission.^2^ Phloem‐feeding by cereal aphids reduces nutrient availability for the plant.^3^ Saprophytic fungal growth on aphid honeydew also lowers photosynthesis efficiency.^4^ Virus transmission, such as the spread of the Barley Yellow Dwarf Virus (BYDV), can lead to wheat yield losses of up to 80%.^1^

Currently, control of aphid infestations is mainly achieved through deployment of broad‐spectrum insecticides.^1^ However, insecticide use is jeopardised due to increasing incidences of insecticide resistance across aphid populations and banning of insecticides, e.g., across the European Union, due to their environmental impacts.^5^, ^6^ This has reduced options that farmers have available to manage pest aphids and highlights the need to identify new approaches to control infestations. New approaches include the use of soil additives,^7^ incorporation of integrated pest management strategies tailored to aphids,^8^ and use of transgenic plants.^9^, ^10^ In this review, we focus on the development of naturally occurring aphid resistance across modern and ancestral wheat lines, which can be engineered into modern elite wheat cultivars, an approach successfully developed against the Russian wheat aphid, *Diuraphis noxia* (Kurdjumov).^11^

**Figure 1:**
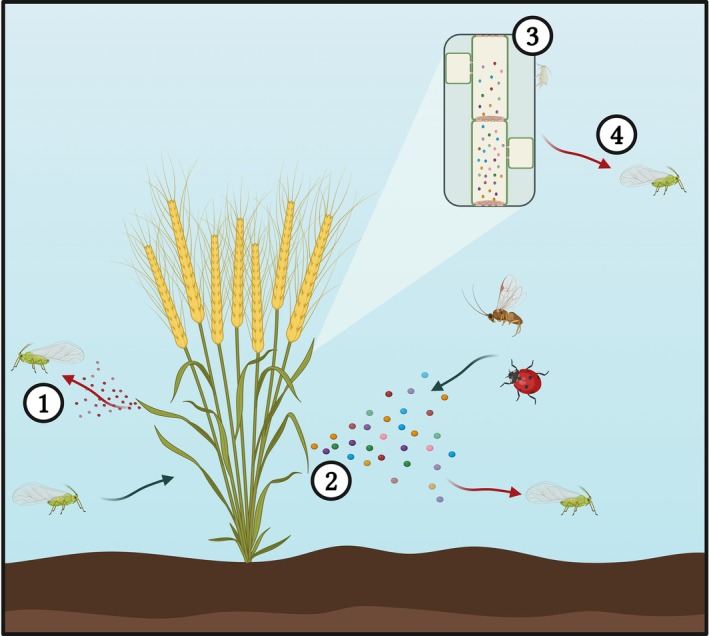
Metabolite‐based aphid resistance mechanisms observed across wheat. 1, constitutively produced volatile organic compounds (VOCs) induce antixenosis towards aphids; 2, aphid‐induced VOCs induce antixenosis towards aphids and attraction towards aphid natural enemies; 3, constitutive or aphid‐induced phloem metabolites induce toxicity towards aphids on feeding (antibiosis); 4, constitutive or aphid‐induced phloem metabolites deter aphids from establishment on host plant (antibiosis). Green arrows = attraction, red arrows = repellence. Created in BioRender. Birkett, M. (2024) BioRender.com/z52z425.

Whilst naturally occurring aphid resistance in wheat can include both physical barriers and metabolite‐based mechanisms,^12^ this review focuses on the latter modes of resistance. Furthermore, host‐plant resistance here is defined by the resistance framework outlined by Stout, namely resistance as plant traits that limit injury to the plant (in contrast to tolerance traits that reduce amount of yield loss per unit injury).^13^ Wheat aphid resistance can be conferred through the release of volatile organic compounds (VOCs) that modify aphid host‐seeking behaviour (antixenosis) or through the presence of non‐volatile primary and secondary metabolites present in the leaves, phloem and roots that modify aphid feeding or development (antibiosis) (Fig. 1). In general, primary metabolites confer resistance by providing reduced amounts of nutrients for aphid development, whilst secondary metabolites act as antifeedants or short‐range deterrents. Secondary metabolites can be constitutively produced or induced by hormonal signalling in response to aphid feeding.^12^ Although classification of metabolites as primary and secondary metabolites is a debated topic,^14^ for simplicity, this nomenclature is used throughout this review. In this case, primary metabolites are classified as those essential for plant growth and reproduction, such as carbohydrates and amino acids, whilst secondary metabolites (also called specialised metabolites) are highly diverse compounds, which may not be directly essential for the plant but play a role in ecological functions, such as defence.^15^

English grain aphids, *Sitobion avenae* (Fabricius), and bird cherry‐oat aphids, *Rhopalosiphum padi* (L.), are the main pest aphids of cereals across the UK and northern Europe, also affecting other major wheat‐growing regions such as South America and China.^1^, ^16^ Both species are vectors for BYDV, which causes yield losses of 5–80%, making it one of the most economically important aphid‐vectored cereal viruses globally.^1^ The use of insecticides to control aphid populations in turn limits the spread of BYDV, which often causes higher yield losses than direct aphid damage.^17^ However, the development of insecticide resistance across aphid populations reduces the efficiency to control against BYDV. Insecticide resistance monitoring across aphid populations has therefore increased in recent years to determine the efficacy of insecticide use in controlling cereal aphids, and in turn BYDV. From across 30 and 29 *S. avenae* and *R. padi* populations monitored in China, respectively, two *S. avenae* and four *R. padi* populations showed high pyrethroid resistance, whilst all populations showed low to moderate neonicotinoid resistance.^18^ Genotypic screening of *S. avenae* populations across Ireland and the UK found the moderately pyrethroid‐resistant SA3 super clone containing the ‘knockdown resistance’ (*kdr*) mutation was dominant in both countries between 2016 and 2018.^6^ Further screening in the UK across 2019–2020 showed moderate pyrethroid resistance in *S. avenae* populations was maintained; however, no signs of resistance was observed in *R. padi*.^19^ The first case of *S. avenae* with the *kdr* mutant providing pyrethroid resistance was identified in northern France in 2021.^20^ More recently, three of 25 sampled *S. avenae* populations from Lower Saxony, Germany, showed similar levels of pyrethroid resistance to *kdr* mutants and the first instance of reduced pyrethroid sensitivity in Germany for *R. padi*.^21^ Current aphid control measures via insecticide use remain effective; however, increased selective pressures induced by pyrethroids following the ban of neonicotinoids in the UK and EU indicates that continual screening for pyrethroid resistance across aphid populations is important to maintain effective aphid control.^19^, ^21^ This also highlights the need to identify alternative aphid and associated virus control measures.

Modern elite wheat varieties with BYDV resistance, such as RGT Wolverine, RGT Grouse and MN‐Washburn, have been developed in recent years, conferring resistance via the ‘*bdv2*’ gene originating from *Thinopyrum intermedium* (Barkworth & D.R. Dewey).^22^, ^23^, ^24^ It works via pathogen‐associated molecular pattern‐triggered immunity and may involve viral movement restriction in the phloem and increased phenolic compound production.^25^, ^26^ Here, we summarise and discuss the current literature surrounding metabolite‐based antixenotic and antibiotic aphid resistance mechanisms of wheat against *S. avenae* and *R. padi*. Current knowledge gaps, and the obstacles and research required to develop aphid‐resistant modern elite wheat are also highlighted.

## 2 VOC‐MEDIATED APHID RESISTANCE IN WHEAT (ANTIXENOSIS)

Aphid host location, as for other pest–host interactions, is mediated by a number of factors, including olfactory (antennal) perception of VOCs produced and emitted by host plants.^27^ Plant VOCs act as attractive or arrestant cues for aphids, or they induce an antixenotic behavioural response. The mechanisms of olfaction‐based aphid host location have been reviewed.^28^ Often involving a complex blend of compounds, attractive VOC cues facilitate host location, arrestant VOCs slow the movement of aphids, whilst antixenotic VOCs reduce the aphid numbers reaching the plant host. Constitutively produced VOCs are typically involved in initial host location and colonisation, suggesting the presence of a suitable host for feeding, whilst herbivore‐induced plant volatiles (HIPVs) released upon aphid feeding typically result in antixenosis (repellence) of other incoming aphids away from plants, indicating the presence of an unsuitable host due to competition for plant nutrients/overcrowding.^29^ By reducing the number of aphids reaching the plant, and thus reducing aphid‐induced damage, antixenosis plays a key role in resistance mechanisms. These can either be constitutive or herbivore induced. VOC‐mediated resistance also includes HIPVs, which can attract aphid predators or prime defences of neighbouring plants, reducing aphid damage indirectly via predation and by bolstering plant defences prior to aphid attack, respectively.^30^

### 2.1 VOC‐mediated aphid resistance in modern and ancestor wheat

A number of studies have confirmed the role of hexaploid wheat VOCs in cereal aphid host location. Behavioural (four‐arm olfactometry) studies confirmed the preference of *R. padi* apterae for a blend of (*Z*)‐3‐hexenyl acetate, (*E*)‐2‐hexenyl acetate, (*E*)‐2‐hexen‐1‐ol, (*Z*)‐2‐hexen‐1‐ol, heptanal, octanal, nonanal, decanal, benzaldehyde and linalool, identified from *T. aestivum* cv. Ciko, and preference of *R. padi* alatae for a blend of (*E*)‐2‐hexenyl acetate, (*Z*)‐3‐hexen‐1‐ol, (*E*)‐2‐hexen‐1‐ol and benzaldehyde.^31^ When tested individually, (*E*)‐2‐hexenal, (*E*)‐2‐hexen‐1‐ol and (*E*)‐3‐hexenyl acetate elicited the same preference.^32^ Interestingly, a similar study assaying the behavioural response of *S. avenae* against (*E*)‐3‐hexenyl acetate, (*Z*)‐3‐hexen‐1‐ol and 1‐hexanol, when tested individually, showed that increasing concentrations of 1‐hexanol caused increased preference, whilst decreased preference occurred with increasing concentrations of (*E*)‐3‐hexenyl acetate and (*Z*)‐3‐hexen‐1‐ol.^33^ Increasing concentrations of all three compounds were accompanied by a stronger preferential response in the aphid predator, the Harlequin ladybird, *Harmonia axyridis* (Pallas), whilst (*Z*)‐3‐hexenyl acetate induced preference in another aphid predator, the hoverfly, *Episyrphus balteatus* (De Geer).^33^ Schröder *et al*.^34^ identified (*Z*)‐3‐hexenyl acetate as an attractant from multiple maize (*Zea mays* (L.)) and wheat cultivars; however, only *Z. mays* cultivar 6Q‐121 induced preference in *R. padi*. Schröder *et al*.^35^ suggested that antixenotic, i.e., repellent, compounds, such as (*E*,*E*)‐α‐farnesene, indole and (*E*)‐2‐hexenal, counteract the preference induced by (*Z*)‐3‐hexenyl acetate in non‐attractive cultivars. These studies show several compounds are common across cereals (Table 1), such as (*Z*)‐3‐hexenyl acetate, and VOC blend composition plays an important role in activity towards aphids, as previously described in the *Aphis fabae* (Scopoli)*–Vicia faba* (L.) aphid–host system by Webster *et al*.^35^ Aphid species and morphs react to different components within the same VOC blend, highlighting the complexity behind VOC‐mediated aphid‐host interactions, which needs to be taken into account when used in integrated pest management (IPM) strategies.

**Table 1:** Summary of common wheat metabolites involved in interactions against *Sitobion avenae* and *Rhopalosiphum padi*, identified from at least two wheat accessions

Compound	Wheat species	Interaction effect compared to controls		References
		*Rhopalosiphum padi*	*Sitobion avenae*	
Volatile organic compounds (VOCs)				
(Z)‐3‐Hexenyl acetate	*Triticum aestivum*	Increased attraction		31, 34, 36
6‐Methyl‐5‐hepten‐2‐one	*Triticum aestivum, Triticum monococcum*	Decreased attraction	Increased and decreased attraction	32, 37, 38, 39
6‐Methyl‐5‐hepten‐2‐ol	*Triticum aestivum*	Decreased attraction	Decreased attraction	32, 37
Linalool	*Triticum aestivum, Triticum monococcum*	Increased and decreased attraction		31, 40
(E)‐2‐Hexen‐1‐ol	*Triticum aestivum*	Increased attraction		31, 32
(E)‐2‐Hexenal	*Triticum aestivum*	Decreased attraction	Increased attraction	32, 34
Heptanal	*Triticum aestivum, Triticum monococcum*	Increased and decreased attraction	Increased and decreased attraction	31, 39, 40
Octanal	*Triticum aestivum Triticum monococcum*	Increased and decreased attraction	Increased and decreased attraction	31, 39, 40, 41
Nonanal	*Triticum aestivum, Triticum monococcum*	Increased attraction	Increased and decreased attraction	31, 36, 39, 41
Decanal	*Triticum aestivum, Triticum monococcum*	Increased and decreased attraction	Increased and decreased attraction	31, 36, 39, 40
Hexadecane	*Triticum aestivum, Triticum monococcum*	Increased attraction	Increased and decreased attraction	31, 39
Heptadecane	*Triticum aestivum, Triticum monococcum*	Increased attraction	Increased and decreased attraction	31, 39
Undecane	*Triticum aestivum, Triticum monococcum*	Increased attraction	Increased and decreased attraction	36, 39
Benzaldehyde	*Triticum aestivum, Triticum monococcum*	Increased attraction	Increased and decreased attraction	31, 39, 41
Leaf secondary metabolites				
HDMBOA‐glucoside	*Triticum aestivum, Triticum durum, Aegilops speltoides*	Deterrent	Deterrent	42, 43, 44
DIMBOA	*Triticum aestivum, Triticum durum, Aegilops speltoides*	Deterrent	Deterrent	42, 43, 44, 45, 46, 47
DIMBOA‐glucoside	*Triticum aestivum, Triticum durum, Aegilops speltoides*	Deterrent	Deterrent	42, 43, 44, 45, 48

High aphid density on wheat and other cereals increases *R. padi* sensitivity to disturbance, reducing aphid preference,^49^ with 6‐methyl‐5‐hepten‐2‐one, 6‐methyl‐5‐hepten‐2‐ol and 2‐tridecanone being responsible for this activity.^37^ These compounds are thought to originate from the aphids, preventing overcrowding on the host plant,^37^ and showed promise for aphid population control in the field on barley (*Hordeum vulgare* L.).^37^, ^50^ Interestingly, 6‐methyl‐5‐hepten‐2‐one and 6‐methyl‐5‐hepten‐2‐ol are HIPVs from the aphid‐resistant *T. aestivum* Beijing837, which induce preference in the aphid parasitoid *Aphidius avenae* (Haliday).^51^ Additionally, saliva‐treated *T. aestivum* Beijing837 induced repellent activity against *S. avenae*, but whether 6‐methyl‐5‐hepten‐2‐one and 6‐methyl‐5‐hepten‐2‐ol are involved in this activity requires confirmation.^38^

Whilst VOC‐mediated resistance in hexaploid wheat has been reported,^38^, ^52^ by contrast, for ancestor tetraploid and diploid wheat, studies directly investigating VOC‐mediated aphid resistance mechanisms are lacking. However, the screening tests mentioned later indicate the presence of potential antixenotic resistance. For example, Liu *et al*.^53^ in choice assays identified *T. turgidum* Lanmai to be less preferred compared to *T. turgidum* Polan305 by *S. avenae*, indicating VOCs may be one of the mechanisms responsible for this activity. Similarly, Elek *et al*.^54^ observed 10 of 12 tested *T. boeoticum* and *T. monococcum* accessions had a reduced number of settled *R. padi* alate compared to modern wheat. The tested diploid accessions also reduced aphid fecundity, indicating non‐VOC‐mediated resistance mechanisms are also present. *T. monococcum* MDR045 and MDR049, previously identified as aphid‐resistant by Elek *et al*.,^54^ showed reduced numbers of *R. padi* and *S. avenae* and increased presence of aphid predators in field trials.^55^ VOC extracts from *R. padi*‐infested and uninfested MDR049 induced antixenotic activity against *R. padi*, with heptanal, octanal, decanal, 4‐ethylbenzaldehyde, 2,4‐dimethyl‐1‐pentene and 4,4‐dimethyl‐2‐pentene reported as potentially responsible for antixenosis.^40^ Similarly, VOC extracts from *S. avenae*‐infested MDR049 and MDR045 induced antixenosis against *S. avenae*, with 21 compounds identified and confirmed as being responsible for this activity.^39^ The same components were identified in aphid‐susceptible *T. monococcum* MDR037 and *T. aestivum* Solstice, indicating compound ratios in VOC blends play an important role in antixenosis,^39^ in line with insect host location theory.^27^

Direct comparison of the efficacy of these antixenotic resistance mechanisms against *S. avenae* and *R. padi* is difficult due to multiple factors, including a lack of data on confirmed antixenosis, use of different assessment methods (ex. olfactometry and choice/settlement assays) and lack of full chemical characterisation of antixenosis. Despite this, some comparisons can be made within assessment types. In two separate studies, *R. padi* spent a similar amount of time (~2–2.5 min) in the treatment arm of a four‐arm olfactometer containing VOCs collected from *T. aestivum* Ciko under *R. padi* damage and *T. monococcum* MDR049 under *R. padi* damage, both being significantly lower than time spent in control arms (~4–4.5 min).^40^, ^56^ VOCs from MDR049 also significantly reduce the time *S. avenae* spend in treatment arms (~1–2 min) compared to controls, which has not been tested for Ciko.^39^ In a separate study, *S. avenae* spent less than 1 min in treated olfactometer arms containing VOCs from an undisclosed *T. aestivum* accession ‘S4’ and *R. padi* spent around 1.7 min in treatment arms containing VOCs from undisclosed *T. aestivum* accessions ‘C3’ and ‘G1’, in both cases significantly less time compared to controls.^57^ This indicates that the antixenotic based resistance mechanisms of MDR049 may be more effective for aphid control because MDR049 is active against both *R. padi* and *S. avenae*, as opposed to Ciko, ‘C3’, ‘G1’ and ‘S4’ acting against single species. Comparing the efficacy of VOC‐mediated antixenotic resistance via choice assays is difficult, as most assess aphid settlement, indicating non‐VOC‐mediated (i.e., contact) resistance, may also influence aphid choices. We could only identify one study which carried out a non‐contact choice assay identifying resistance in wheat against *R. padi*.^52^ In the future, a quantitative analysis of the efficacy of these, and potential synergistic effects of stacked resistance mechanisms, should be carried out to determine the mechanisms that are more likely to provide the best overall control. Despite this, field trials have shown that treatment of barley with methyl salicylate, 6‐methyl‐5‐hepten‐2‐one, 6‐methyl‐5‐hepten‐2‐ol and 2‐tridecanone, formulated as paraffin pellets at 10% w/w, distributed on the soil and replenished every 2 weeks, reduced *R. padi* infestations by 25–50% compared to controls,^50^ showing the potential of VOC‐based control of cereal aphids.

### 2.2 Microbiomes and VOC‐mediated aphid resistance in wheat

**Figure 2:**
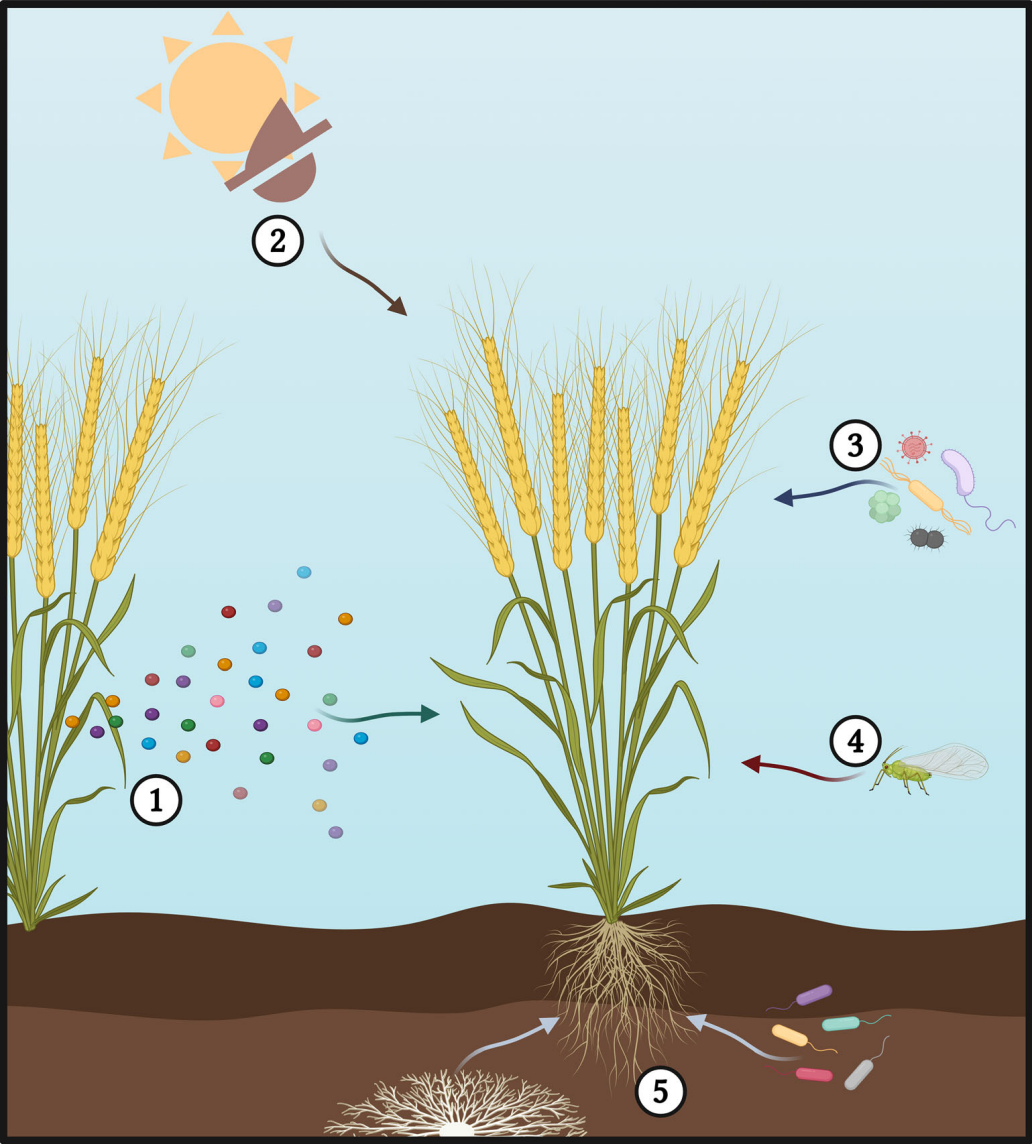
External factors influencing metabolite‐based aphid resistance mechanisms in wheat. 1, herbivore‐induced plant volatiles (HIPVs); 2, abiotic stresses (e.g., heat and drought); 3, aboveground microbial interactions, including beneficial and pathogenic microbes; 4, aphid feeding; 5, microbial rhizosphere interactions. Created in BioRender. Birkett, M. (2024) BioRender.com/I12m481.

Tri‐trophic crop‐aphid‐microbe/virus interactions have also been shown to alter VOC‐mediated aphid resistance in wheat (Fig. 2). VOCs from *Fusarium graminearum* ((Schwein.) Petch)‐infected wheat induced an antixenotic response against *S. avenae* in olfactometry assays, with 2‐pentadecanone and 2‐heptanone as key compounds responsible for this activity.^58^ Conversely, BYDV infection of wheat increased *R. padi* preference compared to uninfected wheat,^36^ with nonanal, (*Z*)‐3‐hexenyl acetate, decanal, an unknown isomer of caryophyllene and undecane identified as the active compounds.^36^ The same study observed that BYDV‐induced preference is lost in viruliferous aphids, which ecologically would facilitate the spread of BYDV to other non‐infected hosts. It should be noted that a similar response has also been documented in wheat‐*S. graminum*‐BYDV interactions.^59^ In contrast, heptanal, octanal, nonanal and decanal were found in higher concentrations in headspace extracts from *T. aestivum* inoculated with endophytic entomopathogenic fungi *Beauveria bassiana* ((Bals.‐Criv.) Vuill.) and *Metarhizium acridum* ((Driver & Milner) J.F. Bisch., Rehner & Humber), eliciting increased preference in both BYDV viruliferous and non‐viruliferous *R. padi*.^60^ A similar preference response was observed in *Myzus persicae* for *Capsicum anuum* (L.) inoculated with either *B. bassiana* or *Akanthomyces muscarius* ((Petch) Spatafora, Kepler & B. Shrestha).^61^

### 2.3 Plant–plant communication and VOC resistance to aphids

In addition to repelling incoming aphids from the plant and attracting natural enemies, aphid‐induced HIPVs play a role in plant–plant communication by ‘priming’ neighbouring undamaged plants for enhanced aphid resistance (Fig. 2). The priming effect of aphid‐induced HIPVs on wheat and other cereals was extensively investigated by Pettersson *et al*.,^49^ showing that defence priming is species‐specific for both aphid and plant host. Variation in priming responses was observed for *S. avenae*, *R. padi* and *M. dirhodum* in barley, oat and wheat hosts. For example, *S. avenae*‐induced wheat HIPVs primed uninfested wheat to produce VOCs inducing an antixenotic response against *S. avenae* but not *R. padi*.^49^ 6‐Methyl‐5‐hepten‐2‐one, in addition to 2‐tridecanone and (*E*)‐2‐hexen‐1‐ol, were shown to prime *T. aestivum* Beijing837 to induce lipoxygenase activity and reduce *S. avenae* feeding and population growth.^62^ These studies indicate that 6‐methyl‐5‐hepten‐2‐one and 6‐methyl‐5‐hepten‐2‐ol play a role in indirect aphid resistance of Beijing837 by priming the plant's jasmonic acid‐mediated defence response and attracting aphid predators. Nonanal, octanal and benzaldehyde are all produced in higher concentrations in mixed *T. aestivum* Florence‐Aurora and Forment cultures, which was attributed to their reduced attractiveness to *S. avenae* when compared to Florence‐Aurora monoculture.^41^ The plant phytohormone *cis*‐jasmone, released as a HIPV, induces wheat VOCs which stimulate preference in the aphid predators *Coccinella septempunctata* (L.) and *A. ervi*.^63^, ^64^

## 3 DEVELOPMENT‐MODIFYING RESISTANCE MECHANISMS (ANTIBIOSIS)

Development‐modifying aphid resistance mechanisms are either metabolite‐based or morphological in nature (such as phloem occlusion). Unlike chewing pests, the specialised feeding mechanism of aphids means they mostly bypass chemical defences in leaf tissues apart from those in the xylem and phloem.^12^ Secondary metabolites are widely known to play a role in plant defence against pests, including aphids. However, primary metabolites also contribute to aphid resistance in wheat.

### 3.1 Primary metabolites

The primary nutritional source for the aphid is host phloem. Although it may not be a direct defence response, reduced phloem sap quality can negatively influence aphid survival, enhancing any direct effects of secondary metabolites. Furthermore, primary metabolites act as feeding stimulants during aphid assessment of host suitability, so reduced levels lower chances of host acceptance.^65^ Primary metabolite, particularly carbohydrate and amino acid, content in the phloem sap of *T. monococcum* MDR049 is lower than that of the aphid‐susceptible *T. aestivum* Solstice and *T. monococcum* MDR037, which is partly attributed to the reduced development and fecundity of *R. padi* on MDR049.^66^ Interestingly, MDR049 showed increased levels of asparagine and glycine on aphid feeding, in addition to threonine and glutamine.^66^ Whether the increase in these amino acids has a direct effect on aphid survival is still unknown; however, they do not outweigh the effects of the reduced carbohydrate and remaining amino acid content observed in MDR049 phloem sap.^66^ Similarly, higher primary metabolite gene expression was observed in the tetraploid *T. turgidum* Zavitan which, in addition to other mechanisms, may contribute to the lack of aphid resistance observed in this accession compared to *T. turgidum* Svevo and *T. aestivum* Chinese Spring.^67^ Effects of abiotic stresses on primary metabolism have gained increased attention because of their effect on aphid survival. Field trials with *T. aestivum* Zhou 22 grown under mild drought conditions showed increased total amino acid concentration, specifically that of arginine, isoleucine, leucine, lysine, glycine and cysteine, which was attributed to increased *S. avenae* abundance and population growth rate.^68^ Conversely, continuous and pulsed drought stress reduced *S. avenae* growth rate and survival in *T. aestivum* Tybalt, which was partly attributed to reduced levels of sucrose and citric acid, increased levels of proline and asparagine, and a relative overall lower concentration of most amino acids in phloem sap.^69^ These studies demonstrate that lower general primary metabolite concentrations are linked to reduced aphid survival. Abiotic stress‐induced changes in primary metabolites have been shown to influence aphid resistance; however, contrasting results indicate that such changes are accession‐specific (Fig. 2). This highlights the need to assess how abiotic stresses influence primary metabolism and subsequent aphid performance. The effects of abiotic stresses may in turn increase the aphid resistance of currently susceptible wheat varieties via reduced phloem sap quality, but this may come at a cost to lower grain quality.

### 3.2 Secondary metabolites

Leaf secondary metabolites have been extensively studied in their role in plant defence against aphids.^12^ Many are often detrimental to plant health in their active form and are thus either stored or transported in an inactive state and become activated on contact with enzymes stored in separate cellular compartments. Activation is facilitated by chewing herbivores, where physical rupturing of leaf cells brings the inactive metabolite and enzyme into contact, creating the active form. Due to their specialised feeding behaviour, aphids mostly bypass secondary metabolite‐associated defence responses, and leaf secondary metabolites involved in aphid defence are only induced on detection of aphid feeding.^12^ The physicochemical properties of secondary metabolites also play a key role in activity: non‐polar compounds are more toxic as they passively cross cellular membranes of the aphid, whilst polar compounds are excreted in honeydew, imparting minimal damage.^70^ Aphids also actively detoxify secondary metabolites.^12^, ^71^, ^72^ The most common leaf secondary metabolites associated with aphid defence responses include alkaloids, cardiac glycosides, benzoxazinoids (BXs) and glucosinolates, all of which have been reviewed.^12^, ^73^

BXs, also known as hydroxamic acids, are the most commonly associated leaf secondary metabolites involved in cereal defence against aphids. BXs are predominantly found in maize and wheat, the most common of which are shown in Table 1. They contribute to (i) resistance against chewing herbivores,^74^ fungal pathogens,^75^ and aphids,^47^, ^76^ (ii) allelochemical activity,^77^, ^78^ (iii) abiotic stress tolerance,^79^, ^80^ and (iv) iron chelation.^81^ Predominantly stored in their inactive glucoside form, BXs are activated by glucosidases on herbivore damage or after ingestion by the pest.^73^ 2,4‐Dihydroxy‐7‐methoxy‐1,4‐benzoxazin‐3‐one (DIMBOA) and its glucoside 2,4‐dihydroxy‐7‐methoxy‐2H‐1,4‐benzoxazin‐3(4H)‐one 2‐O‐glucoside (DIMBOA‐Glc) are the most common BXs in maize; however, the less common 2‐hydroxy‐4,7‐dimethoxy‐1,4‐benzoxazin‐3‐one glucoside (HDMBOA‐Glc) has higher aphid toxicity due to increased instability, spontaneously breaking down into its toxic form in the absence of glucosidases.^82^ The reduced stability of HDMBOA‐Glc results in autotoxicity to the plant and is therefore often only produced on detection of aphid feeding.^82^ The biosynthetic pathway and regulation of BXs have been characterized in maize and partially in wheat,^83^, ^84^ and similarities are observed in herbivore‐induced BX regulation between species.^85^ Wheat BX content is highly variable, high concentrations correlating with higher, albeit partial, aphid resistance.^42^, ^44^, ^45^, ^48^, ^66^ Susceptibility to BXs is aphid species‐specific in *T. turgidum* Svevo, where aphid‐induced DIMBOA and HDMBOA‐Glc elicit resistance to *S. avenae* and partial resistance to *R. padi*, with no resistance to *S. graminum*.^44^ In addition to reducing aphid performance, BXs also show aphid antibiotic properties,^48^ for example high BX content in the wheat‐relative *Aegilops* sp. is attributed to reduced *R. padi* fecundity.^43^ The presence of BXs in *Ae*. *speltoides* (Tasuch.) indicates BX biosynthesis in tetraploid and hexaploid wheat is derived from the B genome. Wheat BX content is influenced by external chemical stimuli, with exposure to *cis*‐jasmone reducing *S. avenae* settling and reproduction and increasing BX levels.^86^, ^87^ Apart from their direct effect on aphid performance, BXs act as signalling molecules, which may contribute to aphid resistance, further emphasizing the blurred line between primary and secondary metabolite functions. For example, DIMBOA plays a signalling role in the induction of callose deposition on aphid feeding.^85^ Furthermore, BXs are involved in the regulation of phenolic compounds linked to aphid resistance, with the overexpression of maize BX *O‐methyltransferases* in wheat accompanied by an increase in phenylpropanoid ferulic acid concentrations.^85^ BXs play a role in shaping both above‐ and belowground microbiomes,^88^ which have been shown to affect aphid resistance (Fig. 2).^89^, ^90^ BXs are not the only leaf secondary metabolites involved in wheat‐derived aphid resistance, as diploid *T. monococcum*, *T. boeticum* and *Ae. longissima* (Schewing. & Muschl.) are resistant against *R. padi* but have non‐detectable levels of BXs.^43^, ^54^ Specifically, the potential involvement of phloem occlusion as an aphid resistance mechanism, in addition to the reduced levels of primary metabolites discussed above, confer resistance in *T. monococcum* MDR049.^66^ However, other leaf secondary metabolites may also play a role in aphid defence of MDR049.

Cereal flavonoids are common antifeedant compounds against chewing herbivores and negatively impact the cereal aphids *S. graminum* and *R. maidis* (Fitch).^91^, ^92^, ^93^ There is some evidence which suggests that the flavonoid and phenolic content of wheat plays a role in *S. avenae* and *R. padi* resistance. Reduced aphid infestation on six bread wheat varieties in the field was accompanied by increased total phenol content.^94^ Similarly, the total phenol and tannin content of *T. aestivum* W0923 was attributed to resistance against *R. padi*.^95^ S. *avenae*‐resistant *T. aestivum* Yongliang No.15 and Ganchun No.18 showed an aphid‐induced increase in total flavonoid content, which was correlated to their antibiotic activity.^96^ The same study showed *S. avenae* feeding on susceptible *T. aestivum* accessions decreased both total phenol and flavonoid content, indicating suppression of plant defence responses. Similarly, *S. avenae* induced an increase in apigenin, luteolin, (+)‐catechin and (−)‐epichatechin content in aphid‐resistant *Triticale* Lamberto, suggesting the increased production of these flavonoids is linked to the accession's aphid resistance.^97^ Neither of these studies directly investigated the effects of flavonoids on aphid survival, and therefore the link between flavonoid/phenol content and aphid resistance, although promising, is correlative and requires confirmation. Similar to primary metabolites, abiotic stresses have been shown to influence both flavonoid and BX production in wheat,^73^, ^98^ which in turn could influence SM‐based aphid antibiotic resistance. How abiotic stresses positively or negatively influence SM‐based aphid antibiotic resistance requires further investigation and may be genotype‐dependent.

## 4 FUTURE PERSPECTIVES AND CONCLUSIONS

The need to transition away from insecticide use in managing cereal aphid populations has spurred an increased interest in screening studies to identify aphid resistance across diploid, tetraploid and hexaploid wheat.^52^, ^54^, ^99^, ^100^, ^101^, ^102^, ^103^, ^104^, ^105^ Despite promising results, further investigation is still required to fully elucidate the resistance mechanisms, a crucial step for the eventual incorporation of these traits into modern elite wheat via either traditional breeding or genetic engineering approaches.

VOC‐mediated antixenosis holds promise as an effective resistance mechanism against *S. avenae* and *R. padi*. However, as VOC blend composition plays a crucial role in the type of activity elicited in aphids, i.e., either attraction or repellence, the complex interaction between blend components and their regulation poses a major challenge for the incorporation of VOC‐mediated aphid resistance into modern wheat. For example, a gene encoding for the aphid alarm pheromone (*E*)‐β‐farnesene was introduced into the aphid‐susceptible wheat line *T. aestivum* Cadenza, which was successful in eliciting antixenotic resistance against *S. avenae*, *R. padi* and *M. dirhodum* and attraction of the aphid parasitoid *A. ervi* under laboratory conditions; however, these activities were not translated into the field.^106^ Conversely, laboratory experiments showed no signs of resistance in Watkins and Gediflux wheat against *S. avenae* and *R. padi*, whilst field trials showed reduced *S. avenae* and *M. dirhodium* numbers in Watkins lines, indicating potential antixenotic based resistance.^103^ Furthermore, in terms of metabolite‐based resistance, it may be difficult to confirm which resistance traits (i.e., VOC‐based or development‐modifying), or combination of traits, are responsible for any observed field results unless plant transformation approaches are used. For example, field trials showed reduced *R. padi* and *S. avenae* numbers on *T. monococcum* MDR049.^55^ Laboratory studies showed that MDR049 contains VOC‐ and aphid development‐based resistance mechanisms, which may both take effect in field conditions.^39^, ^40^, ^66^ Unless transformation approaches are used to silence one of these resistance mechanisms, it would be difficult to assess the effectiveness of individual mechanisms. This is required to understand the molecular mechanisms of resistance, the efficacy of individual resistance mechanisms and identify their optimal combinations. This highlights the need for a more robust assessment of aphid resistance traits using laboratory assays to capture different resistance mechanisms in conjunction with field trials.

### 4.1 Challenges for the implementation of metabolite‐based aphid resistance in wheat

Most studies investigating metabolite‐based aphid resistance are laboratory‐based, with only a few incorporating field trials.^50^, ^55^, ^94^ A more thorough assessment on whether resistance observed in the laboratory translates into the field is thus required because translation is not guaranteed, as observed with transformed wheat producing (*E*)‐β‐farnesene.^106^ Furthermore, external stimuli influence metabolite‐based resistance in synergistic or antagonistic ways. These stimuli may be biotic, such as pest herbivory, plant–plant interactions and microbial interactions, or abiotic, such as heat and drought stress, which are not typically assessed in initial laboratory assays but may be experienced in field settings (Fig. 2). Considering climate change, assessing the effects of abiotic stresses on aphid resistance is important to future‐proof these mechanisms. Examples include (i) an increase in antixenotic activity against *S. avenae* in olfactometry assays by VOCs of *F. graminearum*‐infected wheat,^58^ (ii) an increase in susceptibility to *S. avenae* in *T. monococcum* MDR037 and MDR045 colonised with arbuscular mycorrhizal fungi,^90^ (iii) a reduction in BX‐mediated aphid antibiotic activity on caterpillar herbivory in maize,^85^ and (iv) both an increase and decrease in *S. avenae* survival, dependent on wheat variety, on drought stress.^68^, ^69^ Incorporating multiple aphid resistance strategies into a single variety, i.e., antixenotic and antibiotic mechanisms, may impart stronger aphid resistance and minimise development of resistance in aphid populations, as seen against multiple BYDV vector species, where resistance is aphid species‐specific.^39^, ^40^, ^49^ The efficacy of stacked resistance mechanisms should be tested prior to field implementation to ensure interactions between mechanisms induce synergistic, rather than antagonistic, effects on aphid resistance. Furthermore, the effect of incorporating aphid resistance mechanisms in a single wheat variety on other agronomically important traits, such as yield, biotic and abiotic resistance, needs to be evaluated to ensure economic viability. Identifying external interactions which enhance resistance, such as beneficial microbial interactions, should also be investigated, with a recent study showing arbuscular mycorrhizal fungal colonisation increasing flavonoid, polyphenol and enzyme levels associated with nitrogen metabolism in wheat, aiding in defence against aphid infestation.^107^ Considering the complexity surrounding metabolite‐based aphid resistance, a holistic approach to its implementation should be taken. Several challenges need to be addressed to adopt this approach, including (i) screen wheat varieties to identify novel aphid resistance mechanisms, (ii) identify and confirm secondary metabolites involved in aphid resistance, including their biosynthetic and regulatory pathways, (iii) assess resistance mechanism efficacy across a range of aphid virus vector species, (iv) understand the effect of external biotic and abiotic stimuli on aphid resistance mechanisms, particularly those related to climate change, (v) identify beneficial interactions which enhance aphid resistance, (vi) assess the efficacy of accumulating aphid resistance mechanisms in a single wheat variety, (vii) assess translation of resistance from laboratory to field settings, (viii) assess the effect of resistance mechanisms on agronomically important traits, (ix) determine the efficiency of aphid resistance mechanisms in reducing virus damage, and (x) assess and optimise IPM strategies to achieve optimal aphid‐BYDV management.

### 4.2 Implications of metabolite‐based aphid resistance on BYDV management

The efficiency of aphid resistance, including metabolite‐based resistance, on BYDV management is dependent on the type of resistance mechanism employed by the host plant.^108^, ^109^ To the authors’ knowledge, there have been no studies directly investigating the role of metabolite‐based aphid resistance in wheat in BYDV transmission. Despite this, plant resistance to the vector has been shown to decrease viral incidence in different crop‐virus systems.^110^, ^111^, ^112^ BYDV is inoculated in the phloem during aphid probing and feeding, with increased probing and phloem feeding times directly related to increased virus inoculation.^108^, ^113^ More recently, it was discovered that BYDV transmission may occur during brief intracellular punctures in sieve elements or companion cells of the phloem, prior to phloem feeding.^114^ Therefore, aphid resistance mechanisms which prevent aphid alightment, such as VOC‐based antixenosis, are more likely to be effective in controlling BYDV transmission. VOC‐based antixenosis relies on the presence of a more attractive alternative host in the vicinity for the aphid to choose during host location. Furthermore, constitutively produced VOCs that confer antixenosis may be more efficient for BYDV control than aphid‐induced antixenosis via HIPVs, as aphid feeding is required for HIPV induction, increasing chances for BYDV transmission. Despite this, aphid‐induced HIPV attraction of natural enemies may reduce aphid numbers, thereby reducing BYDV spread. Aphid development‐modifying resistance mechanisms, such as via leaf secondary metabolites, would be less effective for BYDV control, as virus transmission may still occur during initial aphid feeding. In this case, deterrent secondary metabolites may increase BYDV transmission as they facilitate the movement of aphids between hosts, increasing feeding events. Antibiosis via toxic leaf secondary metabolites may be effective for minimising BYDV spread by killing infected aphids prior to migration to a new plant. The signalling role played by some secondary metabolites may also contribute to BYDV control. For example, callose induction via BXs may reduce BYDV transmission/spread by blocking sap flow and preventing phloem feeding.^115^, ^116^ Expression of BX secondary metabolites in wheat is insufficient to impart complete aphid resistance; however, the BX biosynthetic pathway has been extensively elucidated in maize, with orthologs and paralogs of these genes identified in wheat.^117^, ^118^ Investigations into the transcriptional regulation of the BX biosynthetic pathway have been reported in maize and wheat,^80^, ^119^ which will be crucial in the exploitation of this pathway to impart its associated resistance traits. Further investigations are required to identify other secondary metabolite classes, and their biosynthetic pathways, involved in *S. avenae* and *R. padi* resistance. The signalling roles of BXs, particularly in the recruitment of rhizosphere microbes (Fig. 2), should be investigated further within the wheat–aphid system to determine whether particular microbial communities enhance aphid resistance traits. Additionally, the effect of climate‐related abiotic factors on aphid resistance needs further exploration to determine the durability of such traits (Fig. 2). Similar to BXs, flavonoids shape belowground plant‐microbe interactions,^120^ which in turn may affect wheat aphid resistance, an area of research that deserves investigation. This is further compounded by the possible involvement of phenolic compounds in BYDV resistance conferred by ‘*bdv2*’, a gene which has been successfully bred into modern elite wheat.^22^, ^25^

Wheat aphid resistance has been shown to change across development time, often imparting resistance at the seedling stage, which is lost in the adult plant.^40^, ^99^, ^104^ In some cases, resistance has been observed in both seedling and adult stages, such as in *T. monococcum* MDR049.^40^ This variation in resistance has implications on the effectiveness of aphid‐BYDV management in the field. Resistance across the entire crop life cycle is ideal to provide protection throughout the growing season; however, seedling resistance may be preferential for aphid‐BYDV management compared to resistance at later plant stages. There are two main migration periods for *S. avenae* and *R. padi* into the crop: autumn and spring.^121^, ^122^ Resistance at the seedling stage could reduce aphid numbers and BYDV incidence during the first migration into the crop and at a point where the plant is most susceptible.^123^, ^124^, ^125^, ^126^ Aphid and BYDV damage at later stages of crop development has been shown to be less severe on yield.^123^, ^124^, ^125^, ^126^ However, further research is required to assess the temporal changes of identified metabolite‐based aphid resistance mechanisms and their implications in effective aphid‐BYDV management across BYDV strains. Considering the complexities surrounding metabolite‐based aphid resistance mechanisms and their role in BYDV management, a framework must be developed to aid in determining the most appropriate mechanisms to employ.

## 5 CONCLUSIONS

Overall, metabolite‐based resistance mechanisms are major contributing factors to aphid resistance in wheat. Further research is required to fully elucidate the metabolites involved, their biosynthetic pathways and the influence abiotic factors have on these interactions. Furthermore, the availability of national germplasms provides an underutilized resource for the identification of further aphid–resistant wheat accessions and traits. Ongoing research in this field is promising and, in addition to a further understanding of the molecular mechanisms involved in the induction of aphid resistance and suppression of defence responses by aphids, it holds potential for the development of resistant wheat lines against *S. avenae* and *R. padi* to alleviate reliance on the use of insecticides and enhance food security.

## AUTHOR CONTRIBUTIONS

ANB, JV, JCC and MAB were involved in conceptualisation of the manuscript. ANB contributed to writing. JV, JCC and MAB revised and supervised manuscript.

## CONFLICT OF INTEREST

The authors declare there are no competing interests.

## Data Availability

Data sharing is not applicable to this article as no new data were created or analyzed in this study.
